# Semiconducting MOFs on ultraviolet laser-induced graphene with a hierarchical pore architecture for NO_2_ monitoring

**DOI:** 10.1038/s41467-023-38918-3

**Published:** 2023-05-30

**Authors:** Hyeongtae Lim, Hyeokjin Kwon, Hongki Kang, Jae Eun Jang, Hyuk-Jun Kwon

**Affiliations:** 1 Department of Electrical Engineering and Computer Science, DGIST, Daegu, 42988 South Korea; 2 Convergence Research Advanced Centre for Olfaction, DGIST, Daegu, 42988 South Korea

**Keywords:** Sensors and biosensors, Sensors

## Abstract

Due to rapid urbanization worldwide, monitoring the concentration of nitrogen dioxide (NO_2_), which causes cardiovascular and respiratory diseases, has attracted considerable attention. Developing real-time sensors to detect parts-per-billion (ppb)-level NO_2_ remains challenging due to limited sensitivity, response, and recovery characteristics. Herein, we report a hybrid structure of Cu_3_HHTP_2_, 2D semiconducting metal-organic frameworks (MOFs), and laser-induced graphene (LIG) for high-performance NO_2_ sensing. The unique hierarchical pore architecture of LIG@Cu_3_HHTP_2_ promotes mass transport of gas molecules and takes full advantage of the large surface area and porosity of MOFs, enabling highly rapid and sensitive responses to NO_2_. Consequently, LIG@Cu_3_HHTP_2_ shows one of the fastest responses and lowest limit of detection at room temperature compared with state-of-the-art NO_2_ sensors. Additionally, by employing LIG as a growth platform, flexibility and patterning strategies are achieved, which are the main challenges for MOF-based electronic devices. These results provide key insight into applying MOFtronics as high-performance healthcare devices.

## Abstract (web summary)

NO_2_ monitoring is important in urban areas where pollutant levels are typically higher. Here authors present a hybrid structure of laser-induced graphene and Cu_3_HHTP_2_, a 2D semiconducting MOF, for highly sensitive and rapid detection of NO_2_ at the parts-per-billion level.

## Introduction

Safeguarding a livable environment from air pollution is a global challenge due to the rapid pace of urbanization. In particular, NO_2_ accounts for 1.8% of all cardiovascular deaths (e.g., myocardial infarction, heart attack, and diabetes) and even causes degenerative brain diseases such as Parkinson’s disease^1–3^. Considering these negative impacts on the environment and quality of life, the WHO has set the exposure limit for NO_2_ to ~5 ppb (10 µg/m^3^) in the Global Air Quality Guidelines^4^. Accordingly, there is an urgent need to develop technology to monitor NO_2_ at the ppb level in real time to provide personalized pollutant information. To establish a high-performance NO_2_ monitoring system for the prevention of the diseases caused by NO_2_, the following conditions should be satisfied: (1) real-time detection of ppb-level NO_2_, (2) simple operation (no external thermal- or photoexcitation), (3) cost-effectiveness, and (4) wearability (light and flexible). To satisfy these requirements, researchers have developed NO_2_ sensors using various materials, such as metal oxides, transition metal dichalcogenides (TMDs), and carbon-based nanomaterials. However, most conventional materials cannot satisfy the criteria for the exposure limit (~5 ppb) due to low sensitivity at the level of a few ppm^5,6^. In addition, the slow response and recovery times of more than a few minutes were inadequate for real-time monitoring of the concentration of NO_2_^7,8^_,_ and poor reversibility often resulted from the dosimetric behavior of sensors^9,10^. Also, the use of external energy sources (thermal or light) not only complicated the sensor configuration and consumed a considerable amount of energy but also induced low selectivity and baseline drift^6,11,12^.

Thus, 2D semiconducting metal-organic frameworks (MOFs) are a compelling opportunity to fabricate highly sensitive NO_2_ sensors. MOFs, consisting of inorganic secondary building units coordinated with organic linkers, exhibit a designable topology, record-breaking large surface area, and uniform pore size distribution^13^. Recently, with advances in materials science, electrically conductive MOFs have been discovered over the last decade. Therefore, the application of MOFs to electrical sensory platforms has been extensively studied to utilize their superior properties that could favor the adsorption of gaseous species and surface reactions^14,15^. In particular, semiconducting Cu_3_HHTP_2_ (HHTP = 2,3,6,7,10,11-hexahydroxytriphenylene) exhibited exceptional sensitivity and selectivity toward NO_2_ gas as a result of strong redox activity^16–19^. Nevertheless, there are practical challenges associated with using MOFs for NO_2_ sensors owing to limited mass flow and imperfect repeatability^20,21^. Thus, we need a breakthrough to develop MOF-based NO_2_ sensors with rapid and reliable sensing performance.

Here, we introduce laser-induced graphene (LIG) as a growth platform for Cu_3_HHTP_2_ MOFs to accomplish real-time monitoring of ppb-level NO_2_. LIG is an emerging 3D macroporous material that can be produced by direct laser irradiation of various polymers and organic substrates^22,23^. Recently, combining MOFs and graphitic materials, including LIG, has been highlighted in various fields (supercapacitors, batteries, etc.) due to their unique properties^24–28^. However, outstanding gas-sensing performance of the hybrid structure of semiconducting MOFs and LIG has rarely been reported. Through the combination of Cu_3_HHTP_2_ and LIG (denoted LIG@Cu_3_HHTP_2_), we demonstrated a synergistic effect that could not be achieved when MOFs were used alone. First, the nanostructured MOFs grown on LIG enabled accelerated mass transport of the exposed gas due to the lung-mimicking hierarchical macro-/microporous architecture. Furthermore, the increased exposed area maximized the advantage of the MOFs, which have abundant open metal sites and edge ligands to which guest molecules can adsorb. Therefore, the LIG@Cu_3_HHTP_2_ structure exhibited one of the shortest response/recovery times (16 s/15 s) and lowest limit of detection (LoD, 0.168 ppb) among state-of-the-art NO_2_ sensors, even at room temperature and atmospheric conditions. Second, we validated the patterning strategy of solution-based MOF growth, which is one of the most significant challenges in the fabrication of MOF-based electronic devices^15^. As abundant defect sites and functional groups (e.g., -OH) of LIG provide nucleation sites for MOFs, Cu_3_HHTP_2_ could selectively grow on LIG. Finally, MOF-based electronic devices, mostly limited to rigid substrates^29,30^, could be applied to lightweight and flexible substrates through formation on LIG. Therefore, we demonstrated a unique strategy for applying MOFs as personalized wearable sensors.

## Results

### Fabrication of LIG@Cu_3_HHTP_2_

**Fig. 1:**
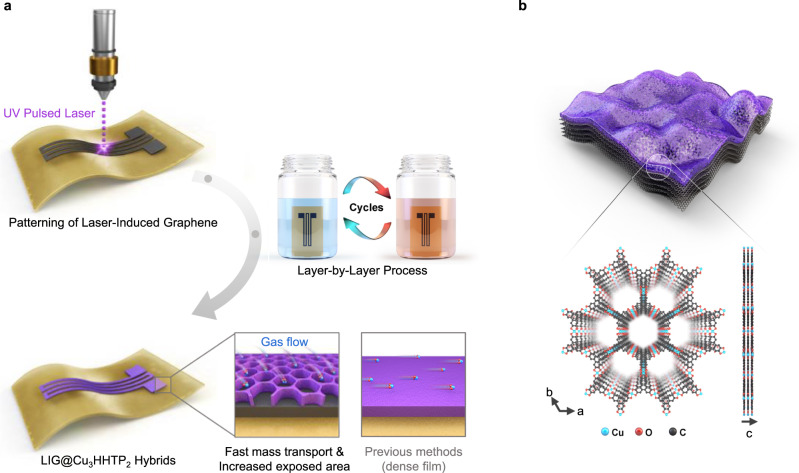
Fabrication of LIG@Cu_3_HHTP_2_. **a** Schematic of LIG@Cu_3_HHTP_2_ processing. Irradiation by the 355-nm laser directly converted PI to LIG. Subsequently, Cu_3_HHTP_2_ MOF is grown on LIG by a layer-by-layer process. **b** Schematic of the structure of Cu_3_HHTP_2_ on LIG.

Figure 1 depicts the fabrication process and a structural schematic of LIG@Cu_3_HHTP_2_. First, we irradiated commercial polyimide (PI) films with a pulsed 355 nm ultraviolet (UV) laser to produce a UV-LIG device (Supplementary Fig. 1). The laser patterning strategy enabled programmable and editable patterning of the electrode compared to conventional photolithography^31^. In addition, unlike previous infrared CO_2_ lasers that photothermally produce graphene, the UV laser directly breaks the chemical bond with intense photon energy at a short wavelength that is strongly absorbed by the PI substrate^32^. Consequently, a UV laser was adopted to miniaturize devices and fabricate thinner substrates, increasing the flexibility of the devices (Supplementary Fig. 2 and Supplementary Note 1).

After fabricating the UV-LIG device, Cu_3_HHTP_2_ MOFs were formed on LIG sheets by a layer-by-layer (LbL) process (Fig. 1a). Considering the mechanical stability of the hybrid materials, the LbL process in which MOFs were grown directly on LIG was selected instead of using unstable traditional transfer methods (drop-casting of a solvothermal solution or spin coating). An optimized LbL process was implemented by repeatedly immersing a UV-LIG device in an ethanolic solution of Cu^2+^ ions and the HHTP ligand at room temperature (Supplementary Fig. 3)^33^. Between immersion steps, UV-LIG was washed with an ethanol reagent. The LbL process is generally accompanied by functionalization of the substrate, such as via piranha treatment, O_2_ plasma, or self-assembled monolayer coating, to create a hydrophilic environment for anchoring of metal ions^34,35^. However, LIG has a chemical environment similar to that of reduced graphene oxides (rGO) with innately abundant dangling bonds and functional groups (-OH, -COOH), thus promoting the nucleation of MOFs without additional functionalization steps^23,24^.

### Analytical characterization of LIG@Cu_3_HHTP_2_

**Fig. 2:**
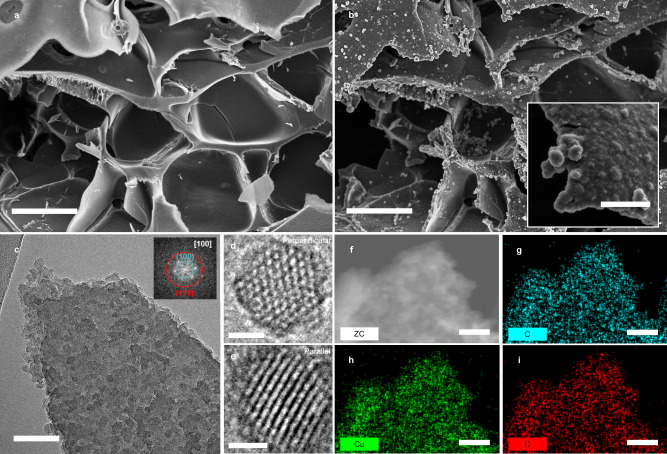
Microscopic analysis of LIG@Cu_3_HHTP_2_. **a** The SEM image of LIG shows its 3D macroporous nature. **b** The SEM image of LIG@Cu_3_HHTP_2_ shows nondestructive growth of Cu_3_HHTP_2_ on LIG. **c** TEM image of LIG@Cu_3_HHTP_2_. The inset shows the FFT of Cu_3_HHTP_2_ in the [100] direction. Enlarged TEM image of Cu_3_HHTP_2_ nanocrystals viewed **d** parallel and **e** perpendicular to the channel. **f**–**i** The EDS-TEM images of Cu_3_HHTP_2_@LIG show uniform distribution of all elements; electron, carbon, oxygen, and copper maps, respectively. Scale bar, 5 µm in **a**, **b**, 500 nm in the inset of **b**, 100 nm in **c**, 10 nm in **d**, **e**, and 100 nm in **f**–**i**.

The scanning electron microscopy (SEM) analysis in Fig. 2a shows the interlaced porous structure of UV-LIG. Numerous macropores originated from the local explosion and release of gaseous species during the photochemical decomposition of the PI substrate^22^. After the laser patterning process, Cu_3_HHTP_2_ was grown on UV-LIG sheets through the LbL process, as shown in the SEM and transmission electron microscopy (TEM) images (Fig. 2b, c). The Cu_3_HHTP_2_ nanocrystals were anchored on the LIG sheets without disruption of the original 3D structure of LIG. The enlarged TEM images of the Cu_3_HHTP_2_ nanocrystals in Fig. 2d, e clearly display hexagonal nanopores and 1D channels with incident electron beams parallel and perpendicular to the channel of Cu_3_HHTP_2_, respectively. The average pore size was 2.02 nm, with a homogeneous size distribution (Supplementary Fig. 4). Well-aligned pores with 1D channels could promote the transport of gas molecules into host–guest interaction sites, such as open metal centers and ligands^35^. As a result of electron microscopy imaging, the incorporation of macroporous LIG and microporous Cu_3_HHTP_2_ to form a hierarchical pore structure was confirmed. The fast-Fourier transform (FFT) image of the [100] direction of Cu_3_HHTP_2_ is shown in the inset of Fig. 2c. The rings of (*hk0*) are clearly observed, while the rings of (*00l*) are missing, implying the alignment of the nanochannels of Cu_3_HHTP_2_^36^. Energy-dispersive X-ray spectroscopy (EDS) images of the Cu_3_HHTP_2_@LIG hybrid (Fig. 2f–i) show uniform distributions of C, O, and Cu, confirming the uniform formation of Cu_3_HHTP_2_ nanocrystals throughout the LIG sheets.

**Fig. 3:**
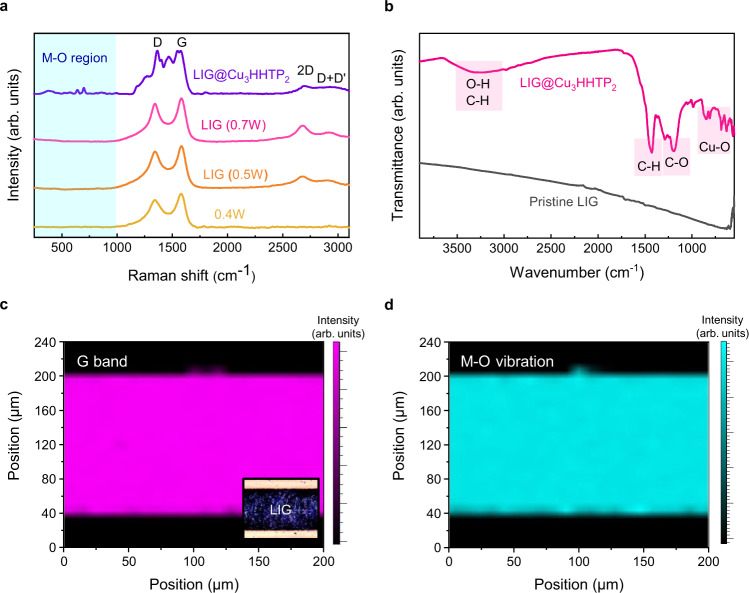
Vibrational spectroscopy analysis of LIG@Cu_3_HHTP_2_. **a** High-resolution Raman spectra and **b** FT-IR spectra of LIG and LIG@Cu_3_HHTP_2_ showing their characteristic vibrational modes. **c** Raman mapping of graphitic G bands in a locally laser-irradiated region. The inset shows an optical microscopic image of the same position. **d** Raman mapping of the M-O vibration mode after the formation of Cu_3_HHTP_2,_ displaying the selective growth of MOF on LIG. Source data are provided as a Source data file.

To further investigate the formation and nature of LIG@Cu_3_HHTP_2_, spectroscopic analyses were performed. Figure 3a shows the high-resolution Raman spectra of UV-LIG fabricated with different laser powers (0.4~0.7 W) and LIG@Cu_3_HHTP_2_. The appearance of G and D peaks indicated the conversion of PI to materials with a high *sp*^2^ carbon content (non-diamond-like materials) upon laser irradiation^37^. Notably, the intense D band (I_D_/I_G_ = ~0.875) was attributed to a high density of edges of the foamy 3D graphene structure and oxidized chemical environment instead of structural defects^38^. As the irradiation power increased (0.5 W or more), the 2D band appeared, which is the second-order overtone of the D band and the fingerprint signal of graphene^39^. Therefore, the formation of few-layer graphene was verified, distinct from amorphous carbon or graphite. The fabrication of LIG@Cu_3_HHTP_2_ was performed at 0.7 W according to the optimized process described in Supplementary Fig. 5. The Raman spectrum of LIG@Cu_3_HHTP_2_ shows the metal–oxygen bond and metal–bis(dioxolane) ring vibration modes of MOFs in low-energy vibrational modes (150–1000 cm^–1^)^40^, the C-H in-plane bending mode of triphenylene (1270 and 1179 cm^–1^), and stretching of the aromatic C-C bonds (1400, 1468, and 1547 cm^–1^)^41^, in addition to the D and G bands. In addition, the constancy of the 2D band indicates that the LbL process is nondestructive to the LIG platform, which is consistent with the results in Fig. 2a, b. Fourier transform infrared (FT-IR) spectroscopy also supports the successful growth of Cu_3_HHTP_2_ on LIG based on the spectral assignment of the vibration modes, as shown in Fig. 3b, Supplementary Fig. 6 and Supplementary Note 2. The broad peak at 3500–3300 cm^–1^ was attributed to C-H/O-H stretching, the strong peak at 1420 cm^–1^ was attributed to C-H bending, the strong peak at 1200 cm^–1^ was attributed to C-O stretching, and the peaks at <1000 cm^–1^ corresponded to M-O stretching modes^19^. The X-ray diffraction (XRD) pattern was recorded for Cu_3_HHTP_2_, as shown in Supplementary Fig. 7. The peaks of Cu_3_HHTP_2_ were in good agreement with previous studies^18,33^.

High-resolution Raman mapping was performed to investigate the selective growth of Cu_3_HHTP_2_ on LIG. Figure 3c shows the spatial mapping of the peaks to baseline values of the graphitic G bands of LIG patterned with a 150 μm line width on the PI substrate. The mapping for the metal–oxygen vibration of the MOFs after the LbL process is shown in Fig. 3d at the same location as in Fig. 3c. The observation of metal–ligand vibration peaks only in the laser-irradiated region means that Cu_3_HHTP_2_ was selectively grown on LIG. The rGO-like chemical environment of LIG provides abundant defect sites and dangling hydroxyl functional groups, which provide an ideal platform for MOFs nucleation by anchoring metal ions^23,24^. Therefore, the selective growth of MOFs along LIG provides a strategy to overcome patterning challenges, which are disadvantages in solution-based processes^29^.

**Fig. 4:**
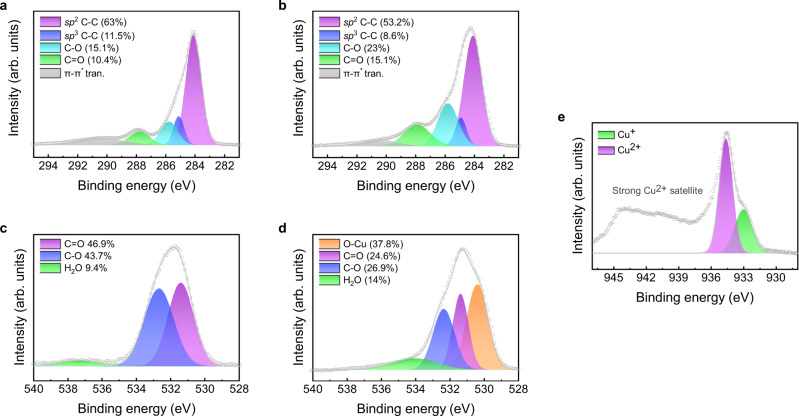
Chemical composition analysis by XPS. **a**, **b** XPS C 1*s* spectra of LIG and LIG@Cu_3_HHTP_2_ hybrids, respectively. **c**, **d** XPS O 1*s* spectra of LIG and LIG@Cu_3_HHTP_2_ hybrids, respectively. **e** XPS Cu 2*p*_3/2_ spectra of LIG@Cu_3_HHTP_2_ hybrids. Source data are provided as a Source data file.

The chemical environment and valence states of LIG@Cu_3_HHTP_2_ were characterized by X-ray photoelectron spectroscopy (XPS, Fig. 4 and Supplementary Tables 1–5). In Fig. 4a, the C 1*s* spectrum of LIG shows a main *sp*^2^ C-C peak (284.1 eV) and a small *sp*^3^ C-C peak (284.8 eV) corresponding to the basal plane and edges of graphene layers, respectively. In addition, the C-O (285.8 eV) and C=O (287.8 eV) peaks observed upon deconvolution confirm that the generated LIG is similar to rGO, hence serving as a nucleation site for MOF, as shown in Fig. 3c, d^24^. After the formation of Cu_3_HHTP_2_ on LIG, the ratio of the C-O and C=O peaks increased in the C 1*s* spectrum (Fig. 4b) because of the catecholate and semiquinonate states of the HHTP ligand^17^. In the O 1*s* spectra (Fig. 4c, d), the O-Cu peak (530.4 eV) could be obviously deconvoluted after the formation of Cu_3_HHTP_2_, which is a result of the coordination of the ligands to the metal centers. The Cu 2*p*_*3*/2_ peak of LIG@Cu_3_HHTP_2_ (Fig. 4e) exhibited an asymmetric shape because a redox-active HHTP ligand capable of having multiple oxidation states exists in the semiquinonate and catecholate states, resulting in Cu^2+^ and Cu^+^ mixed-valency metal states^33^. These characteristics theoretically support the strong sensitivity of Cu_3_HHTP_2,_ which is described in the following section.

### High-performance NO_2_ monitoring of LIG@Cu_3_HHTP_2_

**Fig. 5:**
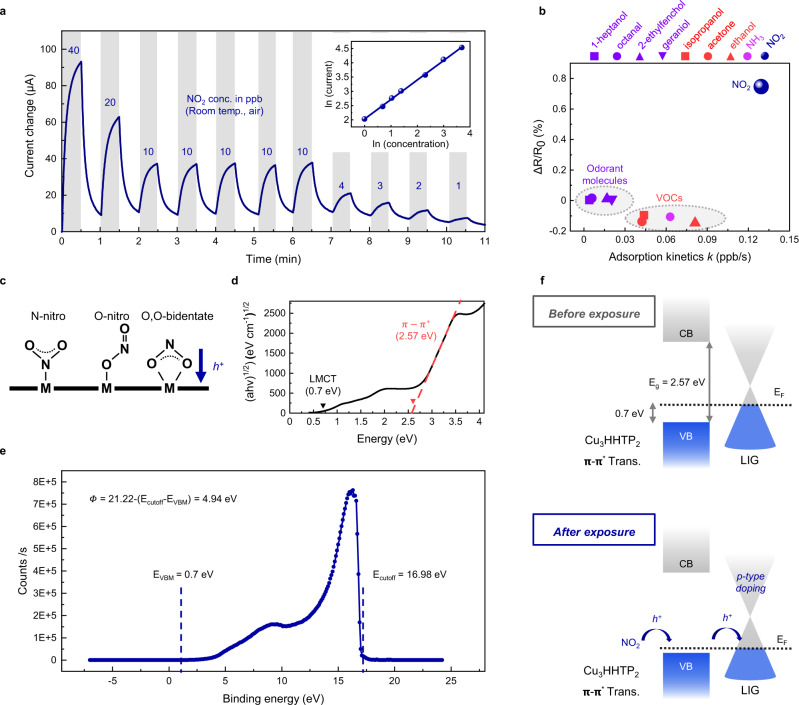
High-performance NO_2_ monitoring by LIG@Cu_3_HHTP_2_. **a** Response and recovery curve with different ppb-level NO_2_ concentrations at room temperature. **b** Selectivity test toward interference gases (NH_3_, VOCs (ethanol, isopropanol, and acetone), and odorant molecules (1-heptanol, 2-ethylfenchol, octanal, and geraniol)). **c** Binding of NO_2_ as a p-type dopant. **d** Tauc plot for bandgap investigation of Cu_3_HHTP_2._ **e** UPS spectrum of Cu_3_HHTP_2_ for deriving the work function and valence band minimum (VBM). **f** Energy band diagram of LIG@Cu_3_HHTP_2_ illustrating p-type doping mechanisms during NO_2_ exposure. Source data are provided as a Source data file.

Real-time monitoring of ppb-level NO_2_ exposure is required for disease prevention and air quality control^42,43^. LIG@Cu_3_HHTP_2_ showed an immediate chemiresistive response and recovery from 40 to 1 ppb NO_2_ without an additional heat or light source (Fig. 5a). In particular, the device showed a sensitive theoretical LoD of 0.168 ppb, which was determined from the threefold noise level and the linear relationship between the current response and the concentration of NO_2_ (inset of Fig. 5a). Furthermore, the selectivity toward NO_2_ over interference gases (volatile organic compounds (VOCs), odorant molecules, and ammonia) in the atmosphere was unambiguously verified by plotting the adsorption kinetic coefficient *k* versus resistance change *ΔR/R*_*0*_ (Fig. 5b, Supplementary Fig. 8 and Supplementary Note 3). Most chemiresistive-type sensors are characterized by poor selectivity^6^. However, LIG@Cu_3_HHTP_2_ showed selectivity to gaseous species by classifying the resistance change and adsorption kinetics. Especially the uniform pore size distribution of MOFs enabled size discrimination of guest molecules, resulting in differences in the adsorption kinetics between gas species^30,44^.

To further investigate the high sensitivity and selectivity of LIG@Cu_3_HHTP_2_, the mechanisms of the interactions between gas molecules and the gas sensor were analyzed. As shown in Supplementary Fig. 9, the sole LIG device showed an insensitive response to NO_2_ without the formation of Cu_3_HHTP_2_. Therefore, fluctuations in the carrier concentration of Cu_3_HHTP_2_ caused by NO_2_ exposure affected the resistance of LIG, enabling the electrical recognition of gas molecules. Note that the main current path of the gas sensor was through LIG, as determined by comparing the I-V curves of LIG and Cu_3_HHTP_2_ (Supplementary Fig. 10). The change in the carrier concentration of Cu_3_HHTP_2_ could be explained by Lewis acid–base reactions between the abundant open metal node and guest molecules. Cu_3_HHTP_2_ has Cu^I^ and Cu^II^ mixed-valency states, as shown in the XPS spectra in Fig. 4. The adsorption of highly acidic NO_2_ gas withdraws electrons from the Cu^I^ metal center, hence coordinating in the form of (1) N-nitro, (2) O-nitrito or (3) O,O-bidentate (Fig. 5c)^16,45^. In contrast, exposure to basic or neutral molecules induces electron donation to Cu^II^ to fill the unoccupied *d* orbital^46^. Accordingly, NO_2_ serves as a p-type dopant that increases the hole carrier concentration of the absorbents. Investigation of the energy band diagram revealed the p-type semiconducting nature of Cu_3_HHTP_2_, as determined by UV‒vis spectroscopy (Fig. 5d) and ultraviolet photoelectron spectroscopy (UPS, Fig. 5e), as shown in Fig. 5f. The process of extracting the band diagram of Cu_3_HHTP_2_ is described in detail in Supplementary Fig. 11 and Supplementary Note 4. Therefore, the p-type dopant NO_2_ increased the majority carrier concentration of Cu_3_HHTP_2_, and the Fermi level also approached the valence band minimum (VBM). To maintain the charge equilibrium between Cu_3_HHTP_2_ and LIG, the work function of LIG increased. Note that LIG exhibits p-type semiconducting behavior upon exposure to gaseous species (Supplementary Fig. 9)^47–49^, meaning that the Fermi level is located in the valence band. Eventually, exposure to NO_2_ increased the majority carrier of LIG and current flow so that it could be sensitively converted into an electrical signal. In addition, bandgap opening of LIG might occur due to functional groups and defects, but it was a negligible effect on this mechanism as a result of investigating the band structure for LIG (Supplementary Fig. 12 and Supplementary Note 5). On the basis of all the evidence above, selectivity to n-type interference gases was also clarified, reducing the current level and inducing a negative response (Fig. 5g and Supplementary Fig. 8).

**Fig. 6:**
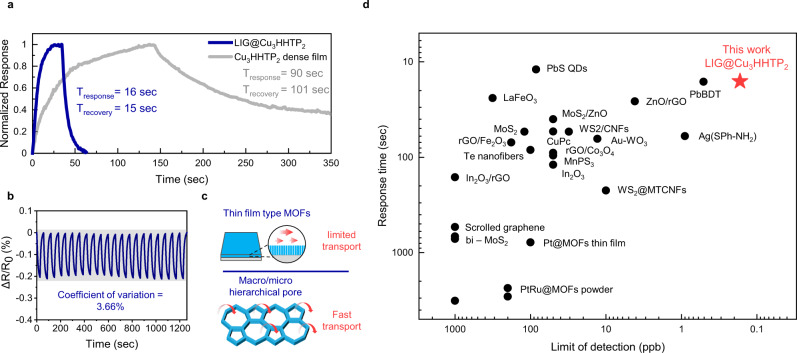
Effect of the hierarchical porous structure. **a** Comparison of the response and recovery time of LIG@Cu_3_HHTP_2_ hybrids and dense Cu_3_HHTP_2_ films toward 10 ppb NO_2_. **b** Repeatability tests with cyclic NO_2_ exposure. **c** Mass transport of the hierarchical porous structure compared to the dense MOF film. **d** Comparison with other state-of-the-art NO_2_ sensing materials operating in air at room temperature in terms of the limit of detection and response time. Source data are provided as a Source data file.

In terms of operation speed, LIG@Cu_3_HHTP_2_ responded very rapidly (16 s for response, 15 s for recovery toward 10 ppb NO_2_, Fig. 6a) compared to most NO_2_ sensing materials, which have response times of more than one minute and exhibit dosimetric behavior that cannot recover completely at room temperature^7–9,17,20^. In addition, as a result of repeated exposure to 10 ppb NO_2_, the device showed perfect recovery and a coefficient of variation (CV) of only 3.66%, as shown in Fig. 6b.

To elucidate the reasons for the fast response and recovery characteristics, we investigated the effect of MOFs formation on a 3D platform. LIG@Cu_3_HHTP_2_ and dense Cu_3_HHTP_2_ films grown on functionalized Si/SiO_2_ wafers were compared, as shown in Fig. 6a, c. The response and recovery times were dramatically reduced in the case of growth on LIG. The promoted time-related characteristics could be inferred from the 3D hierarchical macro/microporous composite structure of LIG@Cu_3_HHTP_2_ (Fig. 6c)^50–52^. In the case of dense Cu_3_HHTP_2_, the limited diffusivity of the film caused a slow response and recovery through the confined nanochannel. The incomplete response and recovery were serious obstacles affecting previous NO_2_ sensing materials^34^. On the other hand, the incorporation of the microporous MOF onto macroporous LIG enabled a large surface area and favored mass transport of gases to active sites (open metal sites or ligands) located in the micropores of Cu_3_HHTP_2_. The human lung structure is an excellent example of a hierarchical pore architecture capable of rapid transport over a large surface area^51^. Therefore, the advantages of MOFs, which have abundant open metal sites and edge ligands that can interact with guest molecules, can be fully utilized. Based on the above mechanisms, to the best of our knowledge, LIG@Cu_3_HHTP_2_ exhibits one of the shortest response times and lowest LoD among state-of-the-art NO_2_ sensing materials (TMD, metal oxides, organic‒inorganic hybrid superlattices, etc.) without heat or light assistance (Fig. 6d)^7,10,17,18,53–69^.

### Stress flexibility tests

**Fig. 7:**
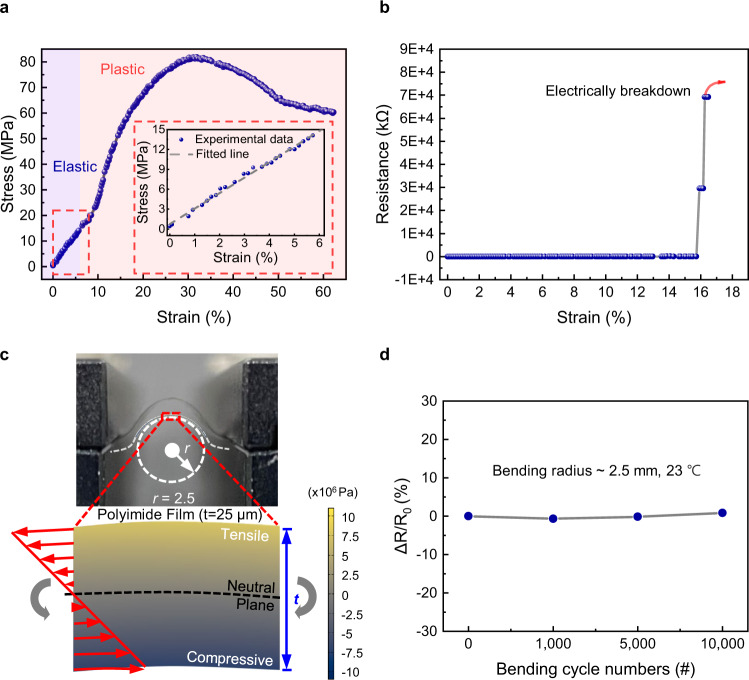
Stress analysis and flexibility tests. **a** Mechanical stress–strain results of the flexible film. **b** Change in electrical resistance during the tensile process. **c** Simulation of the stress distribution of the film by finite element analysis when the radius of curvature is 2.5 mm and the thickness is 25 μm. **d** The fatigue test shows no variation in resistance during the cyclic bending process (*n* = 0, 1000, 5000, and 10,000, when the radius of curvature is 2.5 mm and the thickness is 25 μm). Source data are provided as a Source data file.

Flexibility is essential for reliable sensor operation in a variety of environments, such as wearable platforms and harsh outdoor environments. Therefore, we performed stress analysis and experimented with flexibility based on the design parameters. First, to determine the elastic deformation region of the substrate, a stress‒strain (SS) curve was plotted from the results of a tensile test with 25-μm-thick PI using a universal testing machine, as shown in Fig. 7a. The results showed that the substrate could operate in the elastic region up to ~15 MPa (~6% strain) without mechanical damage, with Young’s modulus of 234 MPa. Furthermore, to determine where LIG@Cu_3_HHTP_2_ was electrically broken down, the electrical resistance was measured in real time during the tensile process, as shown in Fig. 7b. LIG@Cu_3_HHTP_2_ exhibited irreversible electrical breakdown at a strain of ~15.7%, suggesting that the device was electrically stable within the elastic region of the substrate (~6%). The detailed electrical resistance change within the elastic deformation region is shown in Supplementary Fig. 13 and Supplementary Discussion 1.

To optimize the flexible operation, we investigated device parameters such as the radius of curvature and substrate thickness. To determine the range of the bending radius for operation, the stress and strain applied to the PI substrate were calculated with respect to the radius of curvature, as shown in Supplementary Fig. 14a. The substrate underwent elastic deformation up to a bending radius of ~2.1 mm, so the actual bending radius was set to 2.5 mm in consideration of the safety margin. Afterward, Supplementary Fig. 14b shows the calculated stress and strain regarding the thickness of the substrate. Using a PI film with a thickness of less than 35 μm, the device operated in the elastic region at a bending radius of 2.5 mm. Since the excessively thin PI was challenging to handle and the stability of the device may decrease, a 25 μm PI film was selected to achieve fabrication efficiency. Based on the design parameters set in Supplementary Fig. 14a, b, the predicted 2D stress distribution is illustrated in Fig. 7c by using finite element analysis (FEA). The mechanical stress increased farther from the neutral plane, resulting in tensile and compressive stresses of ~10 MPa at the top and bottom, respectively.

To validate the above parameter designs and simulation results, actual bending tests were conducted with a sample with a radius of ~2.5 mm and a thickness of 25 μm. Figure 7d shows the result of the cyclic bending test. Resistance values were measured at the rest position (infinite radius of curvature). Even after 10,000 bending cycles, the device exhibited no significant change in resistance and operated stably without fatigue failure. On the other hand, a change in device resistance occurred at a harsh bending radius of 1 mm (Supplementary Fig. 15), which was predicted to be plastic deformation, as shown in Supplementary Fig. 14a. Consequently, by appropriately setting the thickness and bending radius of the device, the deformation could be elastic without mechanical damage over the top of the film where LIG@Cu_3_HHTP_2_ formed.

## Discussion

In conclusion, we successfully developed a LIG@Cu_3_HHTP_2_ hybrid that could be used to monitor ppb-level NO_2_ in real time. The lung-mimicking hierarchical macro-/microporous structure of LIG@Cu_3_HHTP_2_ improved the mass transport of gas molecules, achieving an immediate response (16 s) and complete recovery (15 s). In addition, by fully exploiting the advantages of MOFs with large surface area and porosity, high sensitivity was achieved due to the increased extent of surface reactions at the active sites (1 ppb experimental minimum detection concentration and 0.168 ppb theoretical LoD). Therefore, LIG@Cu_3_HHTP_2_ exhibited one of the fastest responses and lowest LoD, even without external energy (light or heat source). Furthermore, the uniform pore size of the MOFs allows selectivity according to the adsorption kinetics, adding to the resistance change upon exposure to gases. Such sensitivity and selectivity of LIG@Cu_3_HHTP_2_ are also expected to be useful for effectively designing an artificial olfactory platform.

In terms of device fabrication, the employment of LIG expanded the applicability of MOF-based sensors. The digital laser writing process could produce electrodes without a complex infrastructure, such as that of vacuum equipment, and the selective growth of Cu_3_HHTP_2_ on LIG provided a patterning strategy for MOFs. The UV-laser source also enabled processing on thinner substrates, allowing more flexibility. The flexibility of the sensor was tested by computational simulation and a real cyclic bending test. The sensor endured 10,000 repetitive bending cycles even at a harsh radius of curvature of 2.5 mm. These findings will help guide applications in MOFtronics, which remain at the laboratory level, as a high-performance sensor that could be implemented in the real world.

## Methods

### Materials

All chemicals were obtained from commercial sources and used without further purification. Copper(II) acetate (99.999%) and 2,3,6,7,10,11-hexahydroxytriphenylene (HHTP) ligand (95%) were purchased from Alfa Aesar and Acros Organics, respectively. Kapton PI (thickness: 25 μm) was purchased from the 3M Company.

### Patterning of UV-LIG

LIG electrodes were directly patterned on a PI substrate using a 355 nm ultraviolet pulsed laser (Coherent AVIA-X). The pulse rate and delay time were set to 70,000 Hz and 9.3 μs, respectively. The laser beam was shaped as a line profile to deliver uniform energy to the substrate and decrease the processing time. The scan speed was 5 mm/s, and the laser motion was precisely controlled in the *X-Y-Z-U* directions by an Aerotech stage and actuators. For the formation of LIG@Cu_3_HHTP_2_, the laser power was set to 0.7 W. The power was controlled through a combination of a polarizing beamsplitter and a waveplate. The distance between the objective lens and the sample was controlled by adjusting the height of the Z-stage to maintain the in-focus state. Photographs of the laser setup and irradiation process are shown in Supplementary Fig. 1.

### Cu_3_HHTP_2_ MOF formation by an LbL process

Cu_3_HHTP_2_ MOF was grown on UV-LIG using the LbL process. The PI substrate patterned with LIG was alternatively soaked in an ethanolic solution of 1 mM copper acetate and 0.1 mM 2,3,6,7,10,11-hexahydroxytriphenylene with retention times of 20 and 40 min, respectively. After each soaking cycle, the substrate was washed with ethanol to remove the residual reactants. The trigonal HHTP linker binds to the square planar Cu^2+^ ions to form an extended two-dimensional hexagonal layer in the *ab* plane. Through repeated LbL cycles, MOFs are stacked along the *c*-axis with a 1D open channel. The number of soaking cycles was 8, and the process was accurately automated by using a rotary dip coater (Nadetech ND-R Rotary Dip Coater). The optimization process of the soaking cycles is described in detail in Supplementary Fig. 3. Then, LIG@Cu_3_HHTP_2_ was rinsed with acetone and isopropyl alcohol and dried in a vacuum oven (65 °C, overnight).

### Characterization

The morphologies of LIG@Cu_3_HHTP_2_ were observed by field emission scanning electron microscopy (FE-SEM, Hitachi S-4800 (Fig. 2a, b), and SU8230 (inset of Fig. 2b)). TEM was performed using a Hitachi HF-3300 instrument. For the preparation of TEM samples, LIG@Cu_3_HHTP_2_ was peeled off PI and transferred onto a lacey carbon-supported nickel TEM grid. High-resolution Raman spectra and mapping images were obtained by employing a Renishaw inVia Qontor system using 532 nm laser excitation with a laser power of 5 mW. A Nicolet Continuum infrared microscope (Thermo Scientific) was used to collect the FT-IR spectra. XRD patterns of Cu_3_HHTP_2_ MOFs and LIG were recorded on an Empyrean X-ray diffractometer (Malvern Panalytical) with Cu Kα radiation (λ = 1.54056 Å). XPS and UPS were performed using an ESCALAB 250Xi system (Thermo Scientific).

### Testing of gas-sensing performance

The chemiresistive response was measured on a custom-made gas-sensing test system. Application of DC voltage (1 V) and measurement of current were implemented by a semiconductor analysis system (Keithley 4200). The gas flow was controlled by a mass flow controller (M3030VA, Line Tech). The response and recovery times were defined as the time to reach 90% of the total resistance change. The LoD was determined from Eq. (1) by the IUPAC recommendation.

1 $${{{{{\rm{LoD}}}}}}=\frac{3\,\times {{{{{{\rm{rms}}}}}}}_{{{{{{\rm{noise}}}}}}}}{S}$$

where *S* is the slope of linear fitted data and ${{{{{{\rm{rms}}}}}}}_{{{{{{\rm{noise}}}}}}}$ is the root mean square of noise (standard error). In the response–concentration curve, the slope and standard error were 0.01651 ppb^–1^ and 9.25915 × 10^–4^, respectively. Therefore, the LoD was 3 × (0.01651) / (9.25915 × 10^–4^) ≒ 0.168 ppb. The CV was defined as shown in Eq. (2):

2 $${{{{{\rm{CV}}}}}}=\frac{{R}_{{SD}}}{{R}_{{mean}}}\times 100\%$$

*R*_*SD*_ and *R*_*mean*_ are the standard deviation and mean value of the responses in successive tests, as shown in Fig. 5c.

The response time was defined as the time for the current level to increase from the baseline signal to 90% of the maximum current change. Similarly, the recovery time was defined as the time required for the current level to decrease from the maximum current change to 10% of the maximum current change.

### Flexibility tests

The SS curve and resistance change under the tensile process were measured by means of an SFM-100kN universal testing machine (United Calibration). The resistance change could be recorded in real time by connecting a jig and a digital multimeter (Keithley 2001) with a copper wire. For cyclic bending to test the fatigue failure, a 1-axis motion controller (SCIENCETOWN) was adopted.

## Supplementary information

Supplementary Information

## Data Availability

The authors declare that the data supporting the findings of this study are available within the article and its Supplementary Information files. Additional data are available from the corresponding author upon request. Source Data are provided with this paper.

## Source data

Source Data
