# Supplementary material for: Semiconducting MOFs on ultraviolet laser-induced graphene with a hierarchical pore architecture for NO2 monitoring
Source: Nat Commun. 2023 May 30;14:3114. doi: 10.1038/s41467-023-38918-3 (PMC10229625; doi:10.1038/s41467-023-38918-3)
Supplement: Supplementary file 1 — Supplementary Information [file 41467_2023_38918_MOESM1_ESM.pdf]

Supplementary Information

Semiconducting MOFs on Ultraviolet Laser-Induced Graphene with a Hierarchical Pore Architecture for NO_2 Monitoring

Lim et al.

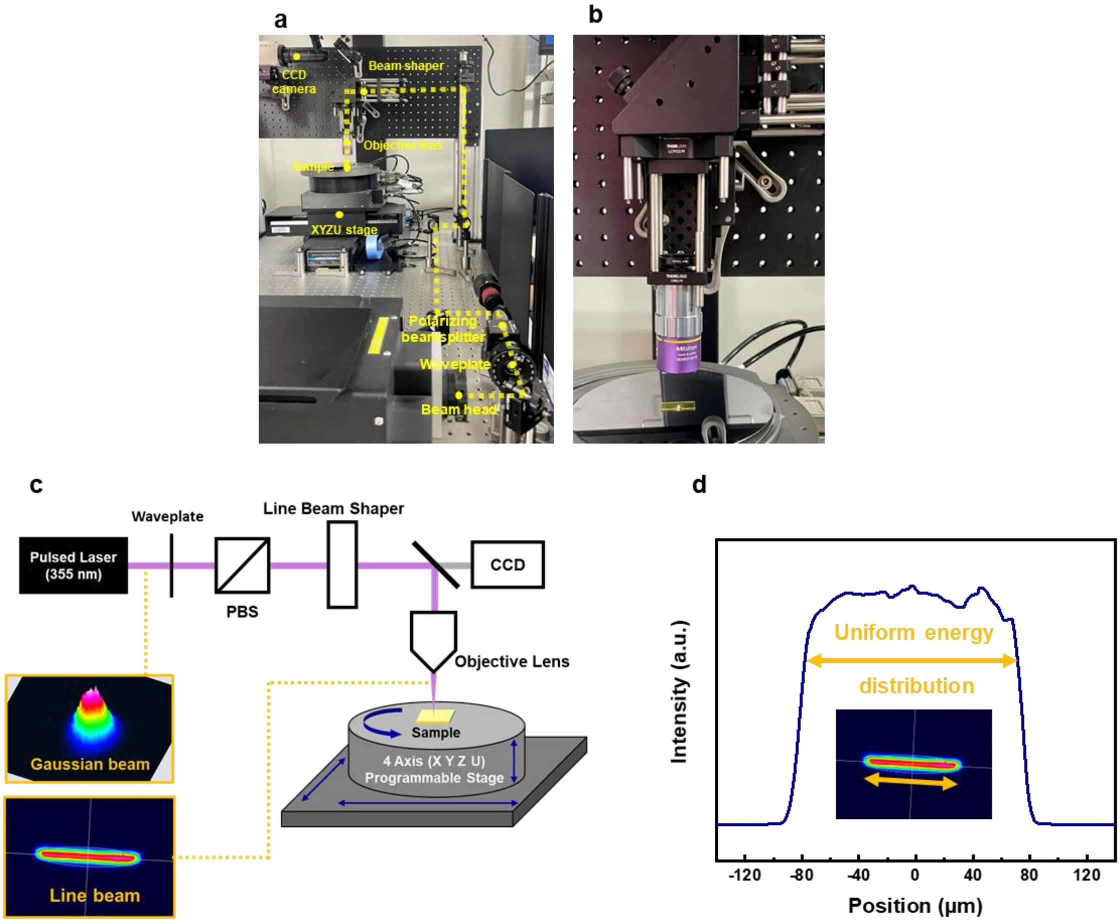

Supplementary Figure 1. a, b Photographs of the laser and the irradiation process. **c** Experimental setting for the 355 nm pulsed laser. **d** Laser intensity distribution plotted parallel to the line profile. The beam profile was measured with a 4M pixels CMOS image sensor. The intensity of initial laser beams has a Gaussian profile, resulting in a difference in energy between the center and the side of the beam. Accordingly, the energy is non-uniformly delivered to the substrate. However, if the laser beam has a line profile through the beam shaper, the energy could be uniformly transferred to the substrate. Therefore, we utilize the line beam through the beam shaper component. In addition, the line beam could be used to irradiate a larger area than a gaussian beam, reducing processing time.

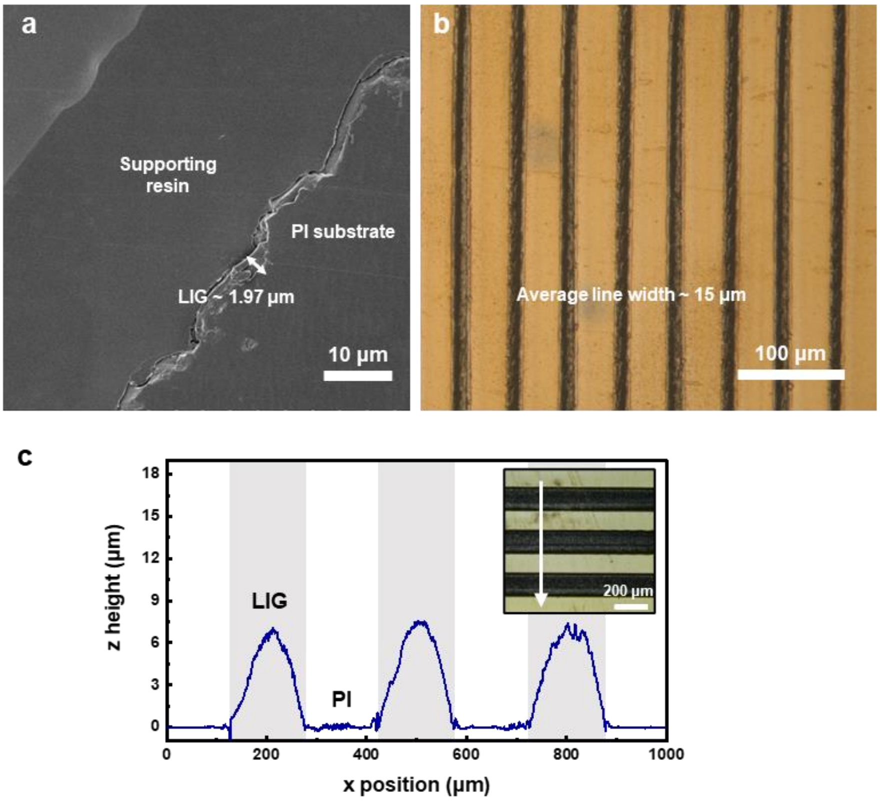

Supplementary Figure 2. Effects of UV-laser irradiation. **a** Cross-sectional SEM image of LIG. A shallow penetration depth without risk of perforation. **b** A minimum patternable line width of 15 μm, dramatically lower than that achieved with irradiation by conventional CO_2 infrared lasers (~100 μm)[1]. **c** Height profile of the laser patterning region measured with an alpha-step stylus.

Supplementary Note 1.

The device used in the actual gas sensing experiment was fabricated using a 150 μm line width. Supplementary Fig. 2b shows that it is also possible to fabricate LIG with a 15 μm line width in a field where device miniaturization is strongly needed. The line width of 15 μm can be obtained without a line beam component. The height profile (Supplementary Fig. 2c) shows geometric information of a sample fabricated with a line beam, which is used in an actual sensing experiment. The local explosion of PI by laser irradiation resulted in the generation of porous LIG protruding from the surface. The cross-section SEM image was obtained using cryo-microtome sampling. Nevertheless, due to damage to the upper part of the soft porous LIG, cross-sectional SEM could not display perfect thickness information for LIG. However, cross-sectional SEM still confirmed that the PI substrate was not damaged, which is the major contributor to mechanical behavior in the *stress flexibility test* part.

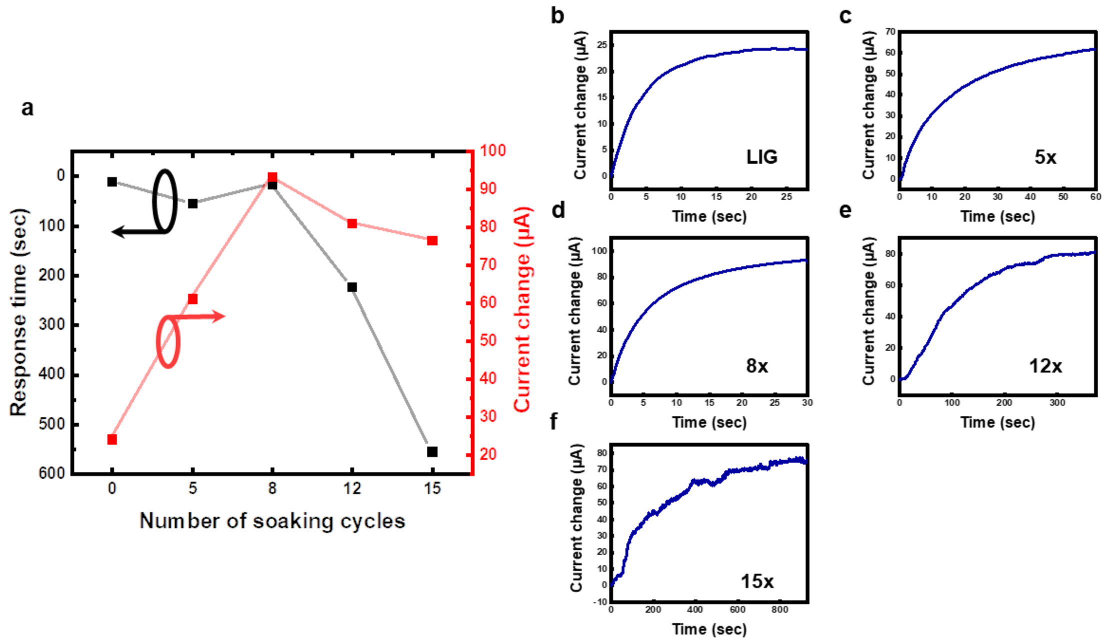

Supplementary Figure 3. Optimization of the LbL process. **a** Response time and current change according to the number of different soaking cycles. **b-f** Response curve toward 100 ppb NO_2 with different numbers of soaking cycles. The optimization process was carried out by comparing the resistance changes and response times for 100 ppb NO_2. A significant current change was observed after a certain number of cycles (8 cycles). The response time also drastically increased over eight cycles. We inferred that excessive cycles made the active MOFs layer too thick, which was unfavorable for the mass flow of gaseous molecules (pore-clogging in LIG). Therefore, the experiment in the manuscript was implemented with eight cycles, considering the reaction time and current change.

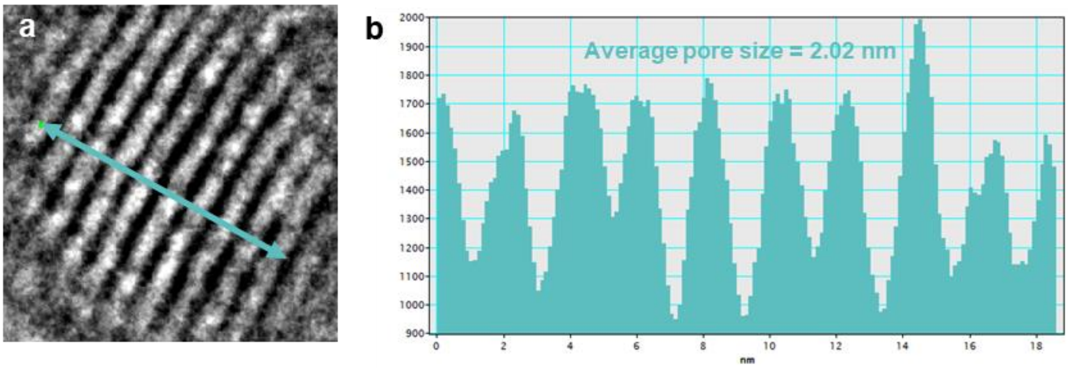

Supplementary Figure 4. Homogeneous pore size of Cu_3HHTP_2 MOFs. **a, b** Intensity of Cu_3HHTP_2 crystal plotted over nine unit cells along the [001] direction, indicating an average pore size of 2.02 nm. This result is consistent with crystallographic data of previous studies[2, 3].

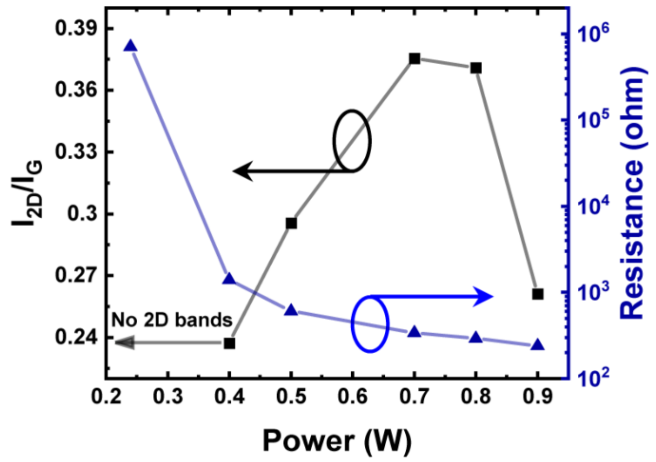

Supplementary Figure 5. Optimization of laser power conditions. The I_{2D}/I_G ratio indicates the quality of the LIG, and the resistance is a major factor in determining the output current level of the chemiresistor type sensor. At a power condition of 0.7 W, the I_{2D}/I_G ratio was the most intensive, and the resistance was saturated. Therefore, laser irradiation for fabrication of LIG@Cu_3HHTP_2 was performed at 0.7 W condition.

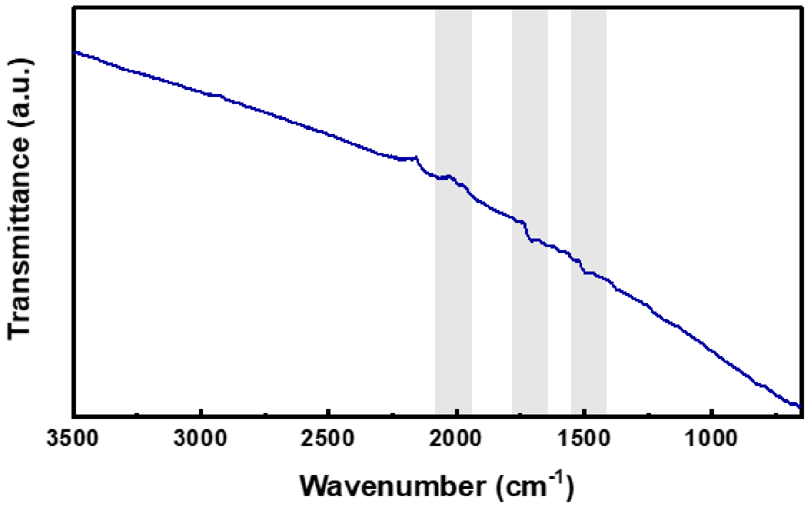

Supplementary Figure 6. FT-IR spectrum of UV-LIG**.**

Supplementary Note 2.

The FT-IR spectra of LIG and LIG@Cu_3HHTP_2 in Fig. 3b cannot clearly show the IR-active modes of pristine LIG. However, the pristine UV-LIG should have IR absorption based on the functional group, as shown in the XPS result in Fig. 4. To provide further information, the enlarged FT-IR spectrum of UV-LIG is attached for reference (Supplementary Fig. 6). UV-LIG shows several IR absorption peaks, including the C=O stretching peak at 1700 cm^{-1}, the C=C stretching peak at 1400-1700 cm^{-1}, and the O-H deformation vibration peak at 1490 cm^{-1}. Nevertheless, compared with those of LIG@Cu_3HHTP_2, the IR absorption peaks of pristine LIG are relatively weak due to the strong IR absorption of MOFs. This phenomenon has been observed in several studies that incorporated MOFs with graphitic materials, demonstrating that the IR absorption of graphitic materials is much weaker than that of MOFs[4-6].

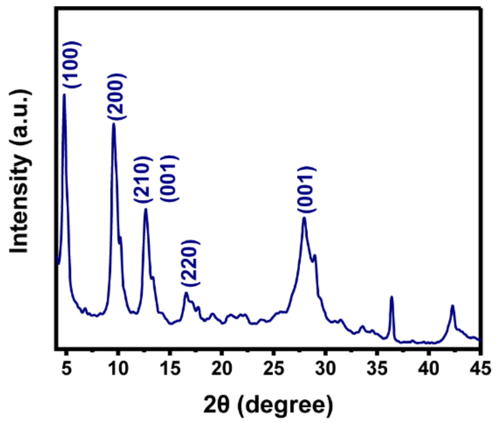

Supplementary Figure 7. X-ray diffraction pattern of Cu_3HHTP_2. The peaks of Cu_3HHTP_2 were in good agreement with previous crystallographic data[2, 7].

Supplementary Table 1. Binding energy, FWHM, and area of the deconvoluted XPS C 1*s* spectrum of LIG.

LIG C 1*s*	Binding energy (eV)	FWHM	Area
sp^2 C-C	281.1	1.232	59393
sp^3 C-C	284.8	0.850	10806
C-O	285.8	1.384	14186
C=O	287.8	1.648	9824
π-π* tran.	290.3	3.687	11606

Supplementary Table 2. Binding energy, FWHM, and area of the deconvoluted XPS C 1*s* spectrum of LIG@Cu_3HHTP_2.

LIG@Cu_3HHTP_2 C 1*s*	Binding energy (eV)	FWHM	Area
sp^2 C-C	281.1	1.446	37837
sp^3 C-C	284.8	0.898	6093
C-O	285.8	1.613	16373
C=O	287.8	2.122	10760
π-π* tran.	290.4	2.731	3923

Supplementary Table 3. Binding energy, FWHM, and area of the deconvoluted XPS O 1*s* spectrum of LIG.

LIG O 1*s*	Binding energy (eV)	FWHM	Area
C=O	531.4	1.767	21505
C-O	532.6	2.024	22932
H_2O	537.34	2.760	2285

Supplementary Table 4. Binding energy, FWHM, and area of the deconvoluted XPS O 1*s* spectrum of LIG@Cu_3HHTP_2.

LIG@Cu_3HHTP_2 O1*s*	Binding energy (eV)	FWHM	Area
O-Cu	530.4	1.467	29668
C=O	531.4	1.075	19325
C-O	532.4	1.467	21103
H_2O	534.1	3.099	8397

Supplementary Table 5. Binding energy, FWHM, and area of the deconvoluted XPS Cu $2p_{3/2}$ spectrum of LIG@Cu_3HHTP_2.

LIG@Cu_3HHTP_2 Cu $2p_{3/2}$	Binding energy (eV)	FWHM	Area
Cu^{+}	933	1.877	13365
Cu^{2+}	934.6	1.457	27291

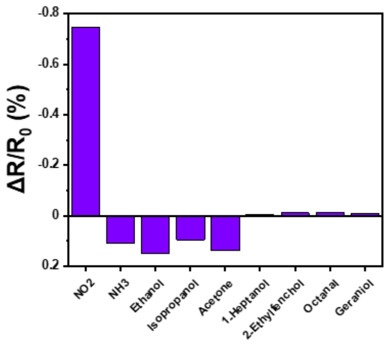

	r_0	k (adsorption kinetics)
NO_2	-0.7454	0.12939
NH_3	0.1067	0.06296
Ethanol	0.1467	0.08119
Isopropanol	0.0955	0.04365
Acetone	0.1383	0.0424
1-Heptanol	-0.0051	0.00389
2-Ethylfenchol	-0.0109	0.01688
Octanal	-0.0145	0.00581
Geraniol	-0.0097	0.02035

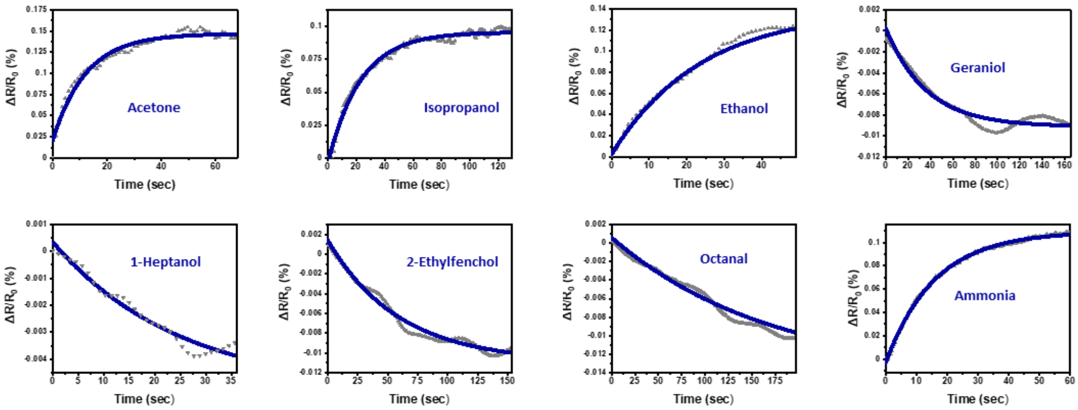

Supplementary Figure 8. Response to interference gases (ethanol, isopropanol, acetone, 1-heptanol, 2-ethylfenchol, octanal, geraniol, and ammonia).

Supplementary Note 3.

In order to confirm selectivity toward interference gases that may exist in the atmosphere, response curves for VOCs (ethanol, isopropanol, and acetone), odorant molecules (1-heptanol, 2-ethylfenchol, octanal, and geraniol) and ammonia were obtained. Based on the pseudo first order reaction model below, adsorption kinetics (k) and response at adsorption-desorption equilibrium (r_0) were extracted.

$$R = A \times e^{-kt} + r_0$$

, where R and A are response and modification factor, respectively.

For weak basic interference gases, LIG@Cu_3HHTP_2 was n-type doped, which induced an increase of r_0. In addition, the odorant molecules with large molecular sizes were also discriminated according to their low k.

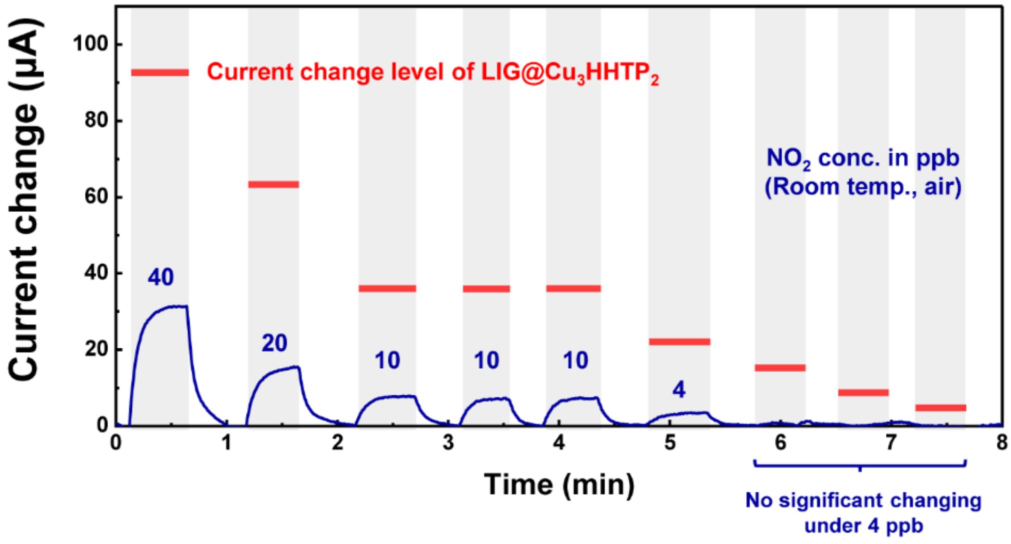

Supplementary Figure 9. NO_2 response and recovery curve of LIG. The LIG device had a smaller response than LIG@Cu_3HHTP_2 (red line) and showed no significant current change under 4 ppb. Note that LIG exhibited *p*-type semiconductive response to NO_2, leading to an increase in current.

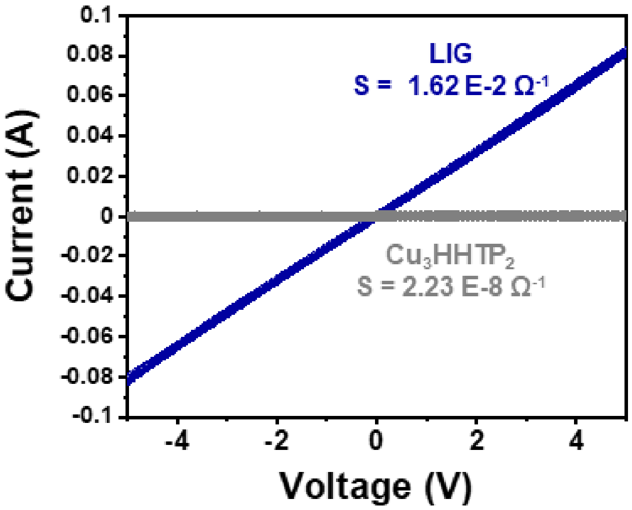

Supplementary Figure 10. I-V characteristics of Cu_3HHTP_2 and LIG. 100 nm Cu_3HHTP_2 thin film was grown on functionalized Si/SiO_2 wafer through the layer-by-layer process. Both LIG and Cu_3HHTP_2 were measured with a channel size of 100 µm * 100 µm. The resultant IV curves indicate that the main current pass of the sensor was LIG.

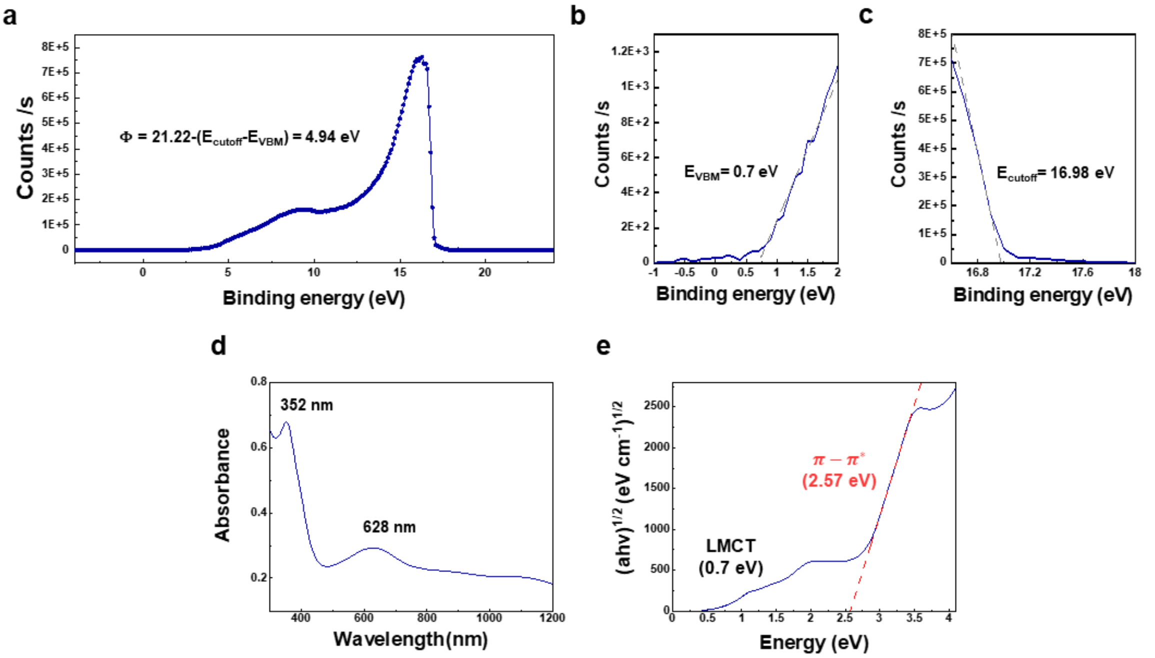

Supplementary Figure 11. Estimation of the energy band diagram of Cu_3HHTP_2. **a-c** UPS spectra and work function calculation of Cu_3HHTP_2. **d** UV–vis absorption spectrum and **e** Tauc plot of Cu_3HHTP_2.

Supplementary Note 4.

The energy structure of Cu_3HHTP_2 was calculated from UPS data and optical bandgap using UV-vis spectroscopy. For both spectroscopy, 50nm Cu_3HHTP_2 films grown on quartz substrates were measured. (a-c) First, fermi level to valence band maximum(E_{VBM}=0.7eV) and workfunction(Φ=4.94eV) of Cu_3HHTP_2 were calculated according to UPS spectrum. (d, e) UV-vis spectra of Cu_3HHTP_2 film show distinct absorption peak at 352 and 628 nm. Then, the two energy states were determined by fitting the Tauc plot[8]. The resulting bandgaps of 0.7eV and 2.57eV are associated with ligand to metal charge transfer (LMCT) and $\pi - \pi^*$transition of the HHTP ligand[9], respectively. Here, because the gas sensing behavior of Cu_3HHTP_2 MOFs has a p-type semiconducting nature experimentally[3], the $\pi - \pi^*$ transition is the major mechanism for gas sensing. Note that the UV–vis spectrum was measured in transmission mode. Also, the UV-vis data almost matched the spectrum of solvothermally synthesized MOFs, meaning that there is no interference from quartz substrate[10].

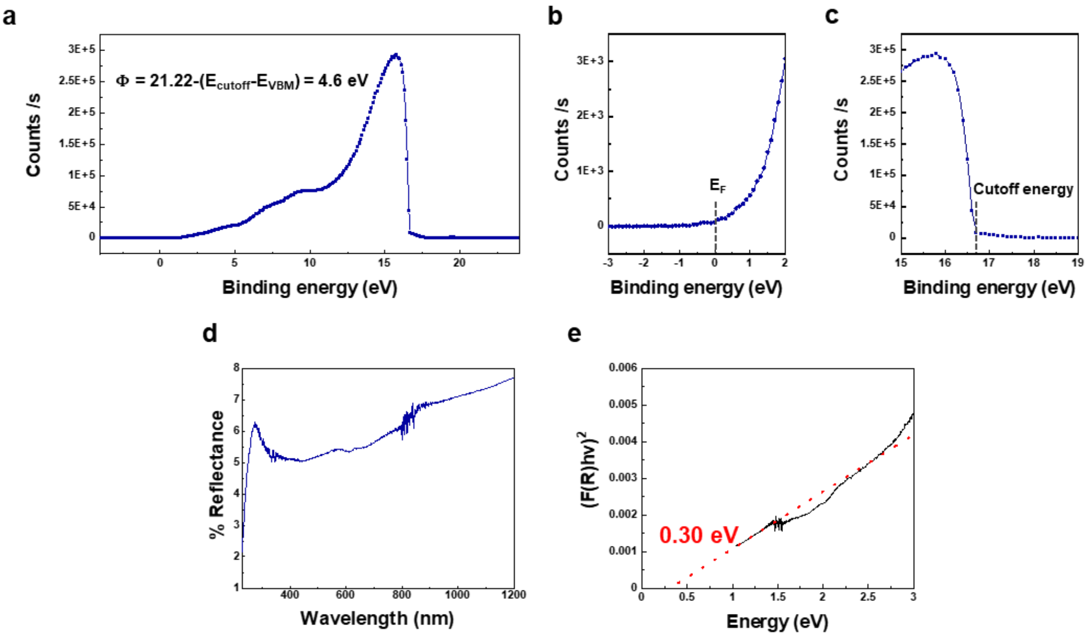

Supplementary Figure 12. Estimation of the energy band diagram of LIG. **a-c** UPS spectra and work function calculation of LIG. **d** UV–vis absorption spectrum and **e** Tauc plot of LIG. The absorption spectrum was obtained by measuring the diffuse reflectance using an integrating sphere.

Supplementary Note 5.

We attached the band structure analysis of LIG for reference in Supplementary Fig. 12. The work function of LIG was calculated to be 4.6 eV, and the UPS signal started at 0 eV binding energy (E_F), indicative of a typical semimetal or narrow-gap material (Supplementary Figure 12a-c). Then, the diffuse reflectance was measured with UV–vis spectroscopy, and the optical bandgap was obtained through the Kubelka-Munk function, which allows for estimation of the bandgap from the diffuse reflectance spectrum[8].

$$F(R) = \frac{(1-R)^2}{2R} \propto \alpha_{K-M} \quad \textbf{(1)}$$

$$(\alpha_{K-M} h\nu)^{(1/n)} = A\left(h\nu - E_g\right) \quad \textbf{(2)}$$

with:

R=reflectance

A=constant

$h\nu$=energy of photon

E_g=optical bandgap

n=2 and 0.5 for allowed indirect and direct transitions, respectively.

The diffuse reflectance spectrum in Supplementary Fig. 12d was converted into the absorption coefficient α_{K-M} using equation (1). Then, equation (2) was used to estimate the optical bandgap of the LIG layer by extending the linear part of the Tauc plot to obtain the y-intercept, as shown in Supplementary Fig. 12e. The optical bandgap was calculated as 0.3eV. In fact, ideal graphene should have zero bandgap Dirac cones, but bandgap openings could occur in LIG due to defects and functional groups. Nevertheless, in view of the high electrical conductivity in LIG and narrow-gap characteristics of the UPS spectrum, the actual electrical bandgap is inferred to be smaller[12, 13]. Therefore, the effect of the bandgap opening of LIG is relatively small compared to the Cu_3HHTP_2 pi-pi* transition bandgap (2.7eV), and we show the bandgap of LIG as zero in Fig. 5.

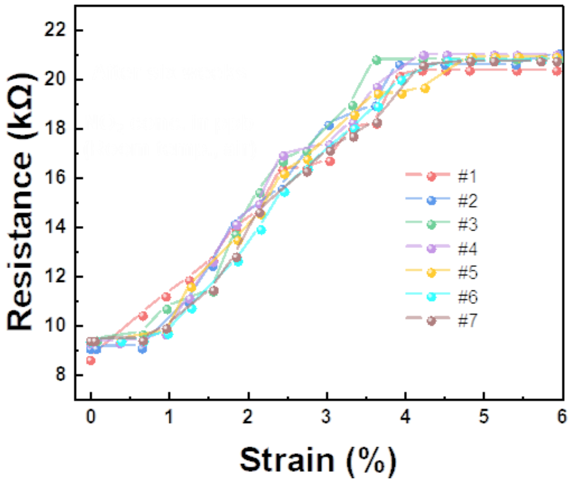

Supplementary Figure 13. Reversible resistance change in the cyclic tensile test in the elastic deformation range of the substrate.

Supplementary Discussion 1.

The current limitations and possible solutions in terms of applying LIG@Cu_3HHTP_2 in flexible electronics are worth noting. In the case of LIG produced on a planar PI substrate without any treatment, the resistance of LIG@Cu_3HHTP_2 reversibly changed during the tensile process in the elastic deformation range due to its piezoresistive property (Supplementary Fig. 13). Accordingly, the device could be applied in a steady-state curvature environment in the current state, but directly utilizing it in applications with load changes in real time (very dynamic and highly strained environments) is challenging. Nevertheless, we envision that this problem will be resolved through mechanically guided structural designs. For instance, fractal-inspired pattern and kirigami strategies are emerging methods to design stretchable electronics, which can effectively decrease local strain[14]. The mechanical guiding strategy can also be readily compatible with the controllable patterning of the laser process. Consequently, the next step for the LIG@Cu_3HHTP_2-based gas sensor will be to increase the stretchability and mechanical robustness for implementation in the real world.

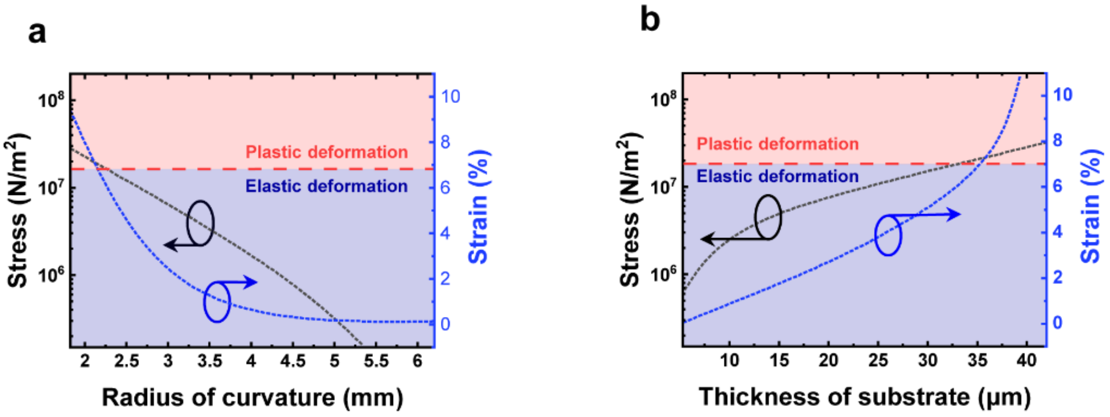

Supplementary Figure 14. Predicted stress and strain curves with respect to various **a** bending radii and **b** thicknesses of the substrate.

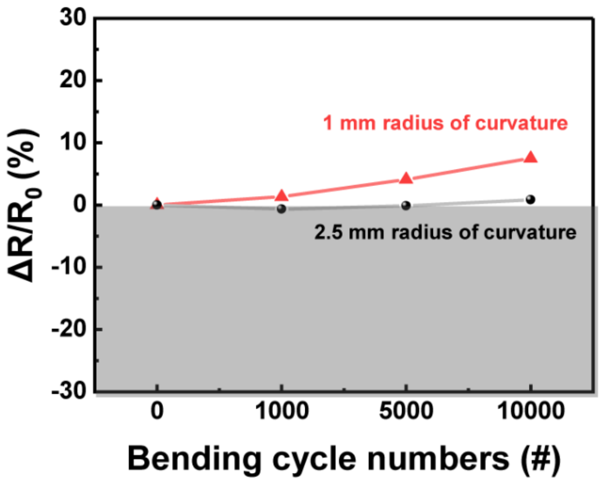

Supplementary Figure 15. Resistance changes in plastic deformation regions (red line) and elastic deformation regions (black line). As a result of the cyclic bending test under a bending radius of 1 mm (red line), a resistance change remarkably occurred.

Supplementary References

1. Carvalho, A.F. et al. Laser-Induced Graphene Strain Sensors Produced by Ultraviolet Irradiation of Polyimide. *Adv Funct Mater* **28**, 1805271 (2018).

2. Nam, K.W., et al. Conductive 2D metal-organic framework for high-performance cathodes in aqueous rechargeable zinc batteries. *Nat Commun* **10**, 1-10 (2019).

3. Yao, M.S., et al. Layer-by-Layer Assembled Conductive Metal-Organic Framework Nanofilms for Room-Temperature Chemiresistive Sensing. *Angew Chem Int Ed Engl* **56**, 16510-16514 (2017).

4. Makhafola, M. D. et al. Palladinized graphene oxide-MOF induced coupling of Volmer and Heyrovsky mechanisms, for the amplification of the electrocatalytic efficiency of hydrogen evolution reaction. *Scientific Reports* **11**, 17219 (2021).

5. Wu, H. et al. Conductive Metal–Organic Frameworks Selectively Grown on Laser-Scribed Graphene for Electrochemical Microsupercapacitors. *Advanced Energy Materials* **9**, 1900482 (2019).

6. Jahan, M., Bao, Q., Yang, J.-X. & Loh, K. P. Structure-directing role of graphene in the synthesis of metal− organic framework nanowire. *Journal of the American Chemical Society* **132**, 14487-14495 (2010).

7. Hoppe, B., et al. Graphene-like metal–organic frameworks: morphology control, optimization of thin film electrical conductivity and fast sensing applications. *CrystEngComm* **20**, 6458-6471 (2018).

8. Tauc, J., Grigorovici, R. & Vancu, A. Optical properties and electronic structure of amorphous germanium. *physica status solidi (b)* **15**, 627-637 (1966).

9. Rubio-Gimenez, V., et al. Bottom-Up Fabrication of Semiconductive Metal-Organic Framework Ultrathin Films. *Adv Mater* **30**, 170491 (2018).

10. Jo, Y.M., et al. Visible-Light-Activated Type II Heterojunction in Cu_3(hexahydroxytriphenylene)$_2$/Fe_2O_3 Hybrids for Reversible NO_2 Sensing: Critical Role of π–π^* Transition. *ACS Cent Sci* **7**, 1176-1182 (2021).

11. Kubelka, P. & Munk, F. An article on optics of paint layers. *Z Tech Phys* **12**, 259-274 (1931).

12. Jin, Y., Zheng, Y., Podkolzin, S.G. & Lee, W. Band gap of reduced graphene oxide tuned by controlling functional groups. *J Mater Chem C* **8**, 4885-4894 (2020).

13. Sutar, D.S., Singh, G. & Divakar Botcha, V. Electronic structure of graphene oxide and reduced graphene oxide monolayers. *Appl Phys Lett* **101**, 103103 (2012).

14. Xue, Z., Song, H., Rogers, J. A., Zhang, Y. & Huang, Y. Mechanically-guided structural designs in stretchable inorganic electronics. Adv. Mater. 32, 1902254 (2020).
